# Supplementary material for: Evaluation of ATOX1 as a Potential Predictive Biomarker for Tetrathiomolybdate Treatment of Breast Cancer Patients with High Risk of Recurrence
Source: Biomedicines. 2021 Dec 12;9(12):1887. doi: 10.3390/biomedicines9121887 (PMC8698757; doi:10.3390/biomedicines9121887)
Supplement: Supplementary file 1 [file biomedicines-09-01887-s001.zip › biomedicines-1321822-supplementary.pdf]

Supplementary material

Table.S1-2; Fig.S1.

Table S1. Demographic table with patient characteristics.

Characteristics	
**Subtype**	
Luminal A and/or B	18
HER luminal	6
TNBC	23
**Tumor stage**	
II	3
III	23
IV	21
**Surgical type**	
Biopsy	14
Mastectomie	14
Lumpectomy	15
CW recurrence	2
primary	1
unknown	1

Table S2. Number of cycles complicated by adverse events (total cycles = 3478)

Adverse Event		N (%)	N (%)
		**All Grades**	**Grade 3/4**
Hematologic			
	Anemia	324 (9.0)	1 (0.03)
	Neutropenia	421 (12.1)	59 (1.7)
	Febrile Neutropenia	1 (0.03)	1 (0.03)
	Leukopenia	427 (12.3)	29 (0.8)
	Thrombocytopenia	31 (0.9)	0 (0)
Gastrointestinal			
	Sulfur Burps	929 (26.7)	0 (0)
	Nausea	76 (2.2)	0 (0)
	Vomiting	8 (0.2)	0 (0)
	Diarrhea	48 (1.4)	0 (0)
	Constipation	7 (0.2)	0 (0)
	Abdominal Pain	1 (0.03)	0 (0)
General			
	Fatigue	798 (22.9)	6 (0.2)
Neurologic			
	Dizziness	26 (0.7)	0 (0)
	Neuropathy	574 (16.5)	5 (0.1)

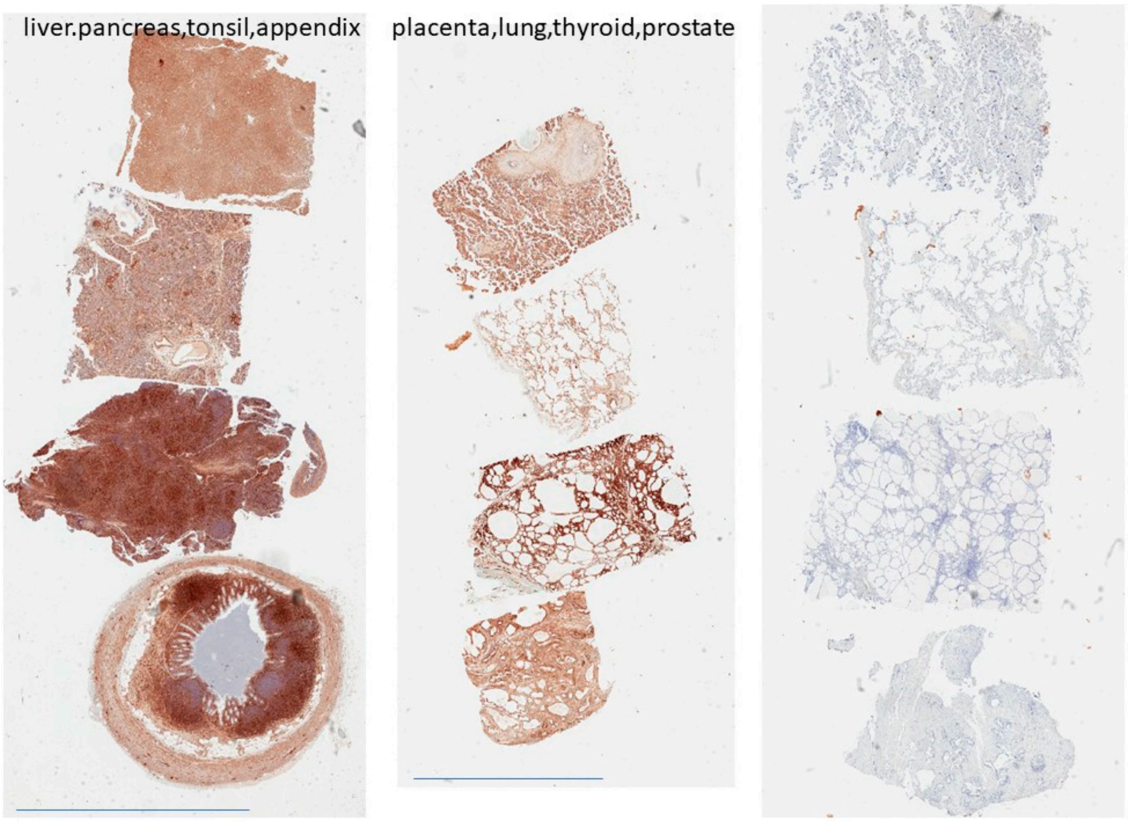

Figure S1. Biological positive (left panel: liver, pancreas, tonsil, appendix; middle panel: placenta lung, thyroid, prostate) and technical negative controls (right panel: placenta, lung thyroid, prostate; technical negative control with omission of primary anti-ATOX1 antibody) for ATOX1 immunohistochemical staining.
